# Isolation and Characterization of *Pectobacterium* Phage vB_PatM_CB7: New Insights into the Genus *Certrevirus*

**DOI:** 10.3390/antibiotics9060352

**Published:** 2020-06-21

**Authors:** Colin Buttimer, Caoimhe Lynch, Hanne Hendrix, Horst Neve, Jean-Paul Noben, Rob Lavigne, Aidan Coffey

**Affiliations:** 1 Department of Biological Sciences, Cork Institute of Technology, T12 P928 Cork, Ireland; colin.buttimer@ucc.ie (C.B.); caoimhe.lynch@mycit.ie (C.L.); 2 APC Microbiome Institute, University College, T12 YT20 Cork, Ireland; 3 Laboratory of Gene Technology, KU Leuven, 3001 Leuven, Belgium; hanne.hendrix@kuleuven.be (H.H.); rob.lavigne@kuleuven.be (R.L.); 4 Department of Microbiology and Biotechnology, Max Rubner-Institut, 24103 Kiel, Germany; horst.neve@mri.bund.de; 5 Biomedical Research Institute and Transnational University Limburg, Hasselt University, 3590 Hasselt, Belgium; jeanpaul.noben@uhasselt.be

**Keywords:** phage, *Certrevirus*, *Vequintavirinae*, homing endonuclease, sigma70 promoter, *Pectobacterium atrosepticum*, phytopathogen, potato blackleg, soft rot disease

## Abstract

To date, *Certrevirus* is one of two genera of bacteriophage (phage), with phages infecting *Pectobacterium atrosepticum*, an economically important phytopathogen that causes potato blackleg and soft rot disease. This study provides a detailed description of *Pectobacterium* phage CB7 (vB_PatM_CB7), which specifically infects *P. atrosepticum*. Host range, morphology, latent period, burst size and stability at different conditions of temperature and pH were examined. Analysis of its genome (142.8 kbp) shows that the phage forms a new species of *Certrevirus,* sharing sequence similarity with other members, highlighting conservation within the genus. Conserved elements include a putative early promoter like that of the *Escherichia coli* sigma70 promoter, which was found to be shared with other genus members. A number of dissimilarities were observed, relating to DNA methylation and nucleotide metabolism. Some members do not have homologues of a cytosine methylase and anaerobic nucleotide reductase subunits NrdD and NrdG, respectively. Furthermore, the genome of CB7 contains one of the largest numbers of homing endonucleases described in a single phage genome in the literature to date, with a total of 23 belonging to the HNH and LAGLIDADG families. Analysis by RT-PCR of the HNH homing endonuclease residing within introns of genes for the large terminase, DNA polymerase, ribonucleotide reductase subunits NrdA and NrdB show that they are splicing competent. Electrospray ionization-tandem mass spectrometry (ESI-MS/MS) was also performed on the virion of CB7, allowing the identification of 26 structural proteins—20 of which were found to be shared with the type phages of the genera of *Vequintavirus* and *Seunavirus.* The results of this study provide greater insights into the phages of the *Certrevirus* genus as well as the subfamily *Vequintavirinae*.

## 1. Introduction

Soft rot *Enterobacteriaceae* (SRE) is a group of economically important phytopathogenic bacteria that consist of the genera of *Pectobacterium* and *Dickeya*, both typified by the production of extracellular pectinolytic enzymes upon plant infection [1,2]. These bacteria cause pre- and post-harvest losses of potato (*Solanum tuberosum* L.) production globally, with this food crop being one of the most intensively grown worldwide [3,4,5]. Of the SRE, *Pectobacterium atrosepticum* has traditionally been the most dominant at affecting the potato crop in temperate climates, causing potato blackleg and soft rot disease [1,6], for which there are currently no effective bactericides. Control strategies used to date mainly consist of culturing practices, including the removal of diseased tissues and/or plants and the implementation of seed certification schemes [7].

Examination of the phages of several well-studied SRE members has shown the potential of phages to be exploited for biocontrol [8,9,10]. The phages of *P. atrosepticum* have also received this attention, with our lab previously demonstrating the capability of a three-component phage mixture to suppress the formation of soft rot caused by this bacterium on potato tubers [11]. Furthermore, Zaczek-Moczydłowska et al. have also demonstrated the ability of a phage cocktail targeting *P. atrosepticum* and *Pectobacterium carotovorum* subsp. *carotovorum* to suppress the formation of potato tuber soft rot and blackleg development in field trials [12].

Currently, there are three types of phages from the order *Caudovirales* known to infect this bacterium. These belong to the subfamilies of *Vequnitavirinae* (a member of the family *Myoviridea)* and *Autographivirinae,* as well as relatives of *Escherichia* virus N4 (the former two groups being members of the family *Podoviridea*) [11,13,14]. Of these groups, two have had genera formally established by the ICTV, namely *Certrevirus* (a member of *Vequintavirus) and Phimunavirus* (a member of *Autographivirinae)* [15]. Currently, the subfamily *Vequintavirinae* contains the genera of *Certrevirus*, *Vequintavirus, Seunavirus* and *Avunavirus* [15]. Before the establishment of *Certrevirus*, members of this group had often been described as rV5 like, due to homology to *Escherichia* phage rV5, the type phage of *Vequintavirus* [14]. The type phage of *Certrevirus* is *Cronobacter* phage CR3 (accession no. JQ691612), the first representative of the *Certrevirus* to have its genome described [16]. Other described phages of this genus include *Pectobacterium* phage ΦTE (accession no. JQ015307) and *Cronobacter* phage PBES 02 (accession no. KT353109), *Cronobacter* phages CR8 (accession no. KC954774) and CR9 (accession no. JQ691611) [14,17]. Furthermore, genomes of *Pectobacterium* phages DU_PP_I (accession no. MF979560) and DU_PP_IV (accession no. MF979563) have become available on public databases, with both of these sharing high pairwise nucleotide identity with phage CR3 (>70%), and possessing similar gene arrangement and should therefore be considered as additional members of this genus. 

In previous publications of our lab, we described phages forming a bank which we are developing against *P. atrosepticum* [11,13]. Here, we report the characterization of another phage in this collection, namely, *Pectobacterium* phage CB7. Phylogenetic analysis of its genome shows that it can be placed within the genus *Certrevirus*. Analysis of phage CB7 provides greater insights into phages of *Certrevirus*, regarding transcription, HNH endonuclease gene splicing, DNA-related metabolism, virion structure, as well as the determination of the suitability of the phage for biocontrol applications. 

## 2. Results

### 2.1. Isolation of CB7, Host Range and General Characteristics

Using *P. atrosepticum strain* DSM 30186 as a host, phage CB7 was isolated from a soil sample collected from potato grading machinery on a farm in Co. Cork, Ireland, in 2013. The phage was differentiated from other phage isolates based on restriction digestion fragmentation patterns of its genomic DNA (Supplementary information 1, Figure S1). Its host range was examined using 31 bacterial strains from five different SRE species, namely *P. atrosepticum* (19 strains), *P. carotovorum* subsp. *carotovorum* (four strains), *Dickeya chrysanthemi* bv. *chrysanthemi* (one strain), *Dickeya dianthicola* (three strains), and *Dickeya solani* (four strains). The phage was found to possess a narrow host range, only exhibiting activity against its host strain *P. atrosepticum* (DSM 30186) and four other strains of the same species (Table 1). Under standard lab conditions, it produced clear plaques with an approximate diameter of 1–2 mm (0.4% LB agar overlay) on its host strain (Supplementary information 1, Figure S2).

**Figure 1:**
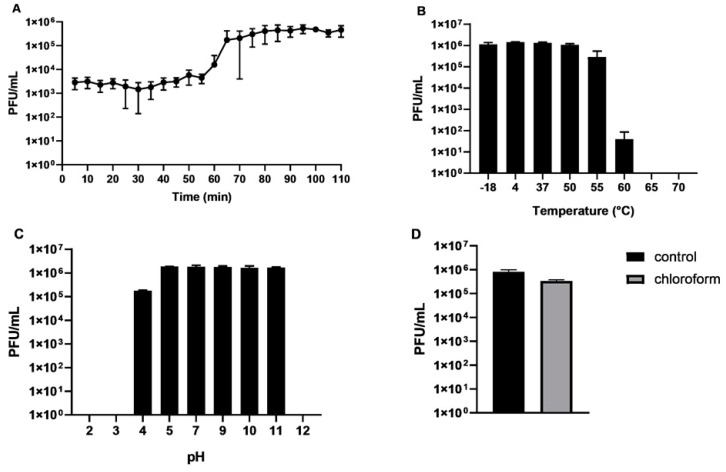
(**A**) Single-step growth curve analysis of *Pectobacterium* phage CB7 on *Pectobacterium atrosepticum* strain DSM 30186 (conducted at 25 °C using LB medium); stability of *Pectobacterium* phage CB7 exposed to (**B**) various temperatures for 1 h and (**C**) various pH conditions for 24 h. (**D**) Stability of *Pectobacterium* phage CB7 after exposure to chloroform (17% v/v) for 1 h.

The one-step growth curve assay, using standard growing conditions with LB medium, showed that CB7 possesses a latent period of 55 min, with an approximate burst size of 154 plaque-forming units (PFU)/cell (Figure 1A). This differs to the CR3-like phage *Cronobacter* phage PBES 02, which has a latent period of 30 min and a burst size of 250 PFU/cell. This isolate is the only representative for which such data is available [17]. The stability of the phage was also examined under different conditions of temperature and pH. Over one hour, the phage was found to be stable between the temperatures of −18 and 55 °C (Figure 1B), and over a period of 24 h was stable between a pH of 5 and 11 (Figure 1C). The temperature stability of CB7 is similar to that of phage PBES 02, but its pH stability range appears to be slightly wider [17]. Additionally, the phage was found to tolerate chloroform after an hour of exposure without a significant log-fold decrease in phage titre (Figure 1D).

**Figure 2:**
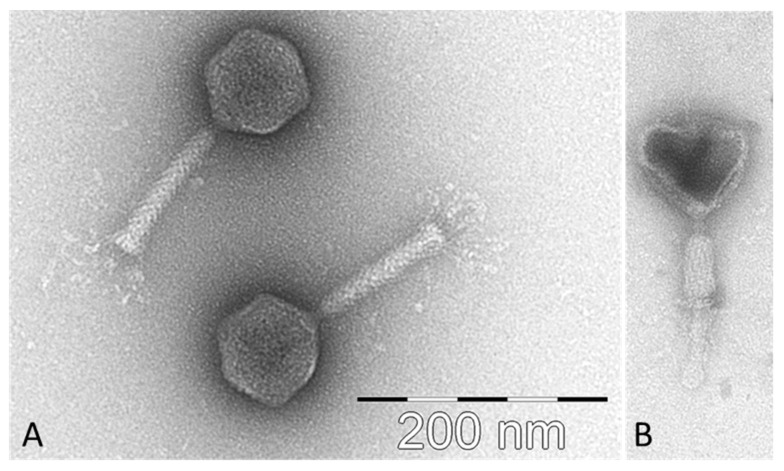
Transmission electron micrographs of *Pectobacterium atrosepticum* phage vB_PatM_CB7 stained with 1% (w/v) uranyl acetate. (**A**) Two uncontracted phage CB7 virion particles and (**B**) one particle with empty and collapsed capsid and contracted tail sheath. Scale bar represents 200 nm.

Transmission electron microscopy showed that CB7 possesses a myovirus A1 morphotype (Figure 2), allowing it to be classified as a member of the order *Caudovirales* [18]. The phage possesses an icosahedral head (84.1 ± 3.9 nm in diameter, *n* = 11), with clearly distinguishable hexagonal outlines, and a contractile tail (123.4 ± 2.6 nm × 20.2 ± 1.1 nm, *n* = 8), possessing a bundle of thin and flexible tail fibres (length: 42.4 ± 3.7 nm, *n* = 6). The phage was formally named in accordance with the nomenclature set out by Kropinski et al. [19].

### 2.2. General Genome Characteristics of CB7

Genome sequencing of CB7 revealed a size of 142,778 bp (coverage 2554×) and examination of sequence reads indicated that its genome is likely circularly permuted like that of coliphage T4 [20] with no region of the genome possessing significantly higher read depths compared to the average read depth across the entire genome. BAL-31 exonuclease time course treatment of the genome followed by restriction digestion with BgII agrees with the above finding, with no bands found to be specifically degraded following examination by agarose gel electrophoresis (Supplementary information 1, Figure S3). A similar result was obtained with the closely related *Pectobacterium* phage ΦTE [14]. Therefore, since the CB7 genome is believed to be circularly permuted, the start position of its GenBank file was matched to that of *Cronobacter* phage CR3. Additionally, the G+C content of its genome was found to be 50.1%, a value similar to what would be typically expected for its host species, *P. atrosepticum* (50–51%) [21,22].

**Figure 3:**
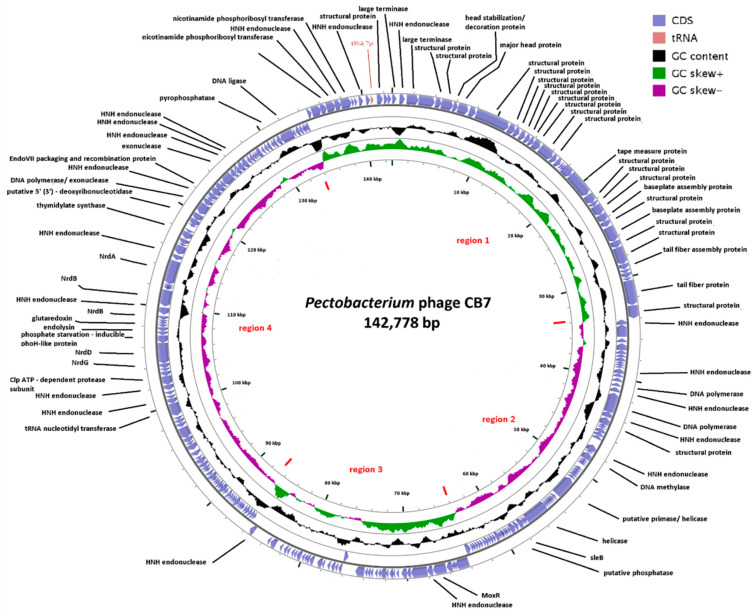
Summary of the genomic organization of the 142,778 bp genome of *Pectobacterium* phage CB7. On the outer ring, putative ORFs are represented by purple arrowheads, labelled with a predicted function where possible. The middle ring shows GC content relative to the mean GC content of the genome. On the inner circle, GC skew is illustrated where green represents positive skew and purple a negative skew. Created using GCView [25].

The genome of CB7 was found to harbor 253 open reading frames (ORFs) (Figure 3 and Supplementary information 2, Table S1). A search of these proteins against the Prokaryotic Virus Orthologous Groups (pVOGs) database could assign 203 to orthologous groups. Based on the orientation of these ORFs, the genome can be divided into four regions, namely region 1 (CB7_241–37), region 2 (CB7_38–103), region 3 (CB7_104–143) and region 4 (CB7_144–240). ORF orientation correlates well with GC skew [23]. Using in silico analysis based on protein sequence similarity, protein structure homology, lipoprotein and transmembrane analysis, and as well as electrospray ionization-tandem mass spectrometry (ESI-MS/MS), it was possible to identify a role for 81 (32%) of these putative proteins. The remaining proteins could be categorized as either hypothetical proteins (9), conserved hypothetical proteins (143), putative lipoproteins (3), conserved (4) and hypothetical (13) transmembrane proteins. Additionally, one tRNA gene for tyrosine was identified. No ORFs were identified to encode integrase, excisionase nor repressor proteins, indicating that the phage likely follows an exclusively lytic lifestyle. Screening against the CARD database [24] indicates that the genome encodes no gene products associated with antibiotic resistance.

### 2.3. Phylogenetic Analysis—CB7 is A Member of Certrevirus and the Status of Vequintavirinae

**Figure 4:**
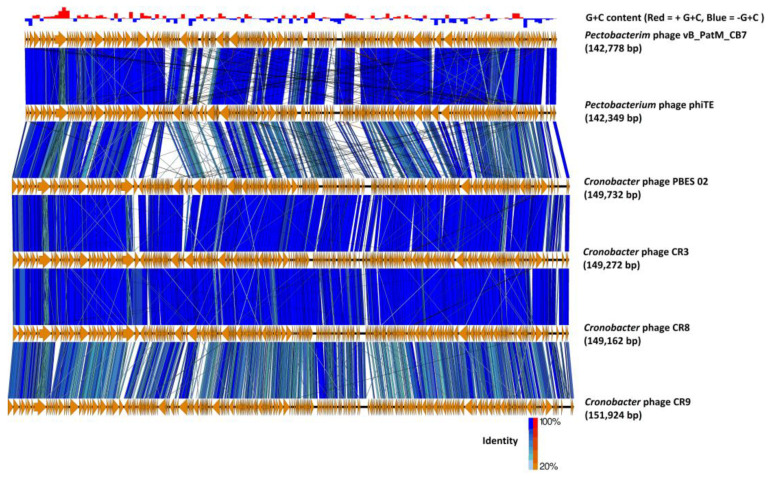
Comparison of the genome of *Pectobacterium* phage CB7 to other CR3-like phages (*Pectobacterium* phage ΦTE and *Cronobacter* phages PBES 02, CR3, CR8 and CR9) using currently available annotations employing TBLASTX and visualized with EasyFig. The bar chart shows the G+C skew of the CB7 genome. Orange arrows indicate locations of genes among the different phage genomes; and lines between genome maps indicate the level of identity (blue/turquoise, genes sharing orientation; red/orange, genes with inverted orientation). The large terminase was set as the first gene among all genomes.

**Figure 5:**
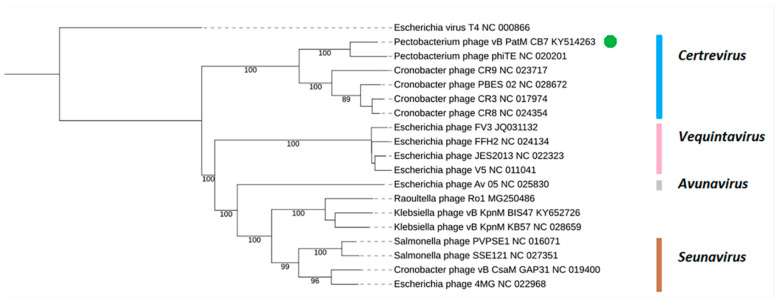
Amino acid VICTOR-generated phylogenomic GBDP tree of *Pectobacterium* phage CB7 (highlighted with a green dot) and 17 members of the *Vequintavirinae* subfamily inferred using the formula D4 and yielding an average support of 84%. The numbers above branches are GBDP pseudo-bootstrap support values from 100 replications. Members of the genera of *Avunavirus*, *Seunavirus*, *Vequintavirus* and *Certrevirus* are illustrated. *Escherichia* phage T4 was used as an outliner.

**Figure 6:**
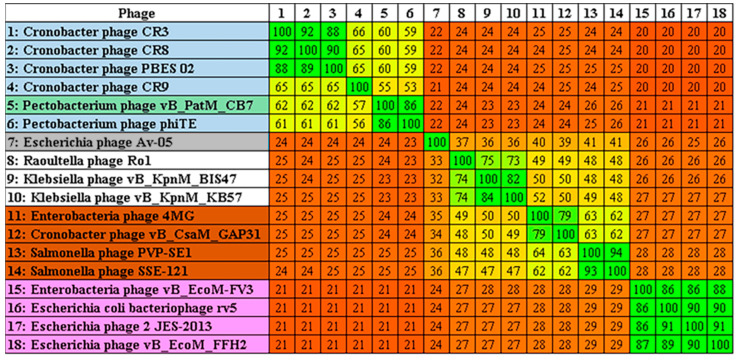
TBLASTX heat map generated using Gegenees with accurate parameters—fragment length: 200 bp; step size: 100 bp; threshold: 0%. The map includes the genomes of *Pectobacterium* phage CB7 (highlighted in turquoise) and 17 members of the *Vequintavirinae* subfamily. Members of the genera *Avunavirus* (grey), *Seunavirus* (brown), *Certrevirus* (blue), and *Vequintavirus* (pink) are illustrated.

Examination of the genome of CB7 using BLASTn showed that the phage has significant sequence similarity with phages of the genus *Certrevirus* (≥60% identity) and shares a similar arrangement of genes (Table 2, Figure 4). CoreGenes analysis also showed a large number of shared proteins (Table 2), confirming CB7 to be a member of the genus *Certrevirus*. Furthermore, construction of phylograms using the large terminase, major head and DNA polymerase proteins of phages within the subfamily of *Vequintavirinae* placed CB7 within a clade that represents the genus of *Certrevirus* (Supplementary information 1, Figure S4). This was further supported by the creation of a GBDP phylogram using VICTOR (Figure 5) and Gegenees analysis (TBLASTX), where CB7 was shown to possess 55% to 86% identity within a clade representing *Certrevirus* (Figure 6).

Analysis performed in this work showed that there are currently eight phage genomes in the public databases that can be classified as *Certrevirus.* New additions to the genus other than CB7, are *Pectobacterium* phages DU_PP_I and DU_PP_IV (Table 2). Generally, phages within the *Certrevirus* genus possess genome sizes that range between 142,349 (φTE) and 151,924 bp (CR9) and possess an average G+C content of 50 ± 0.34%. Total ORF number ranges from 242 (φTE) to 281 (CR9), with CoreGenes indicating that they share a minimum of 166 proteins with phage CR3. However, there is a large variation in tRNA gene content among these phages. Those infecting *Cronobacter* possess between fourteen (PBES 02) and eighteen (CR3) tRNA genes, whereas those infecting *Pectobacterium* encode only one (CB7) to eight (DU_PP_I and DU_PP_IV) tRNA genes.

### 2.4. Transcription, Promoters and Terminators

Phage CB7 was found not to contain a gene encoding an RNA polymerase, suggesting that it is totally dependent on host-encoded RNA polymerase for transcription. Thus, it is suspected that the phage likely possesses genes that play a role in the takeover of host RNA polymerase, causing its redirection to host promoters, as seen in *Escherichia* phage T4 [26].

**Figure 7:**
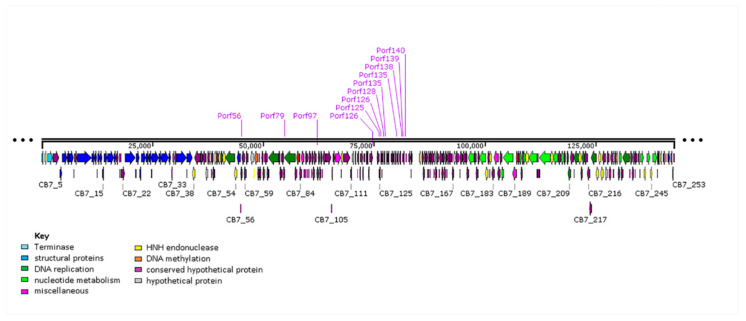
Genome map of *Pectobacterium* phage CB7 showing locations of a putative sigma70-like promoter with the consensus sequence AAAA(N4)TGTTGAC(N17)TATAAT. The map comprises arrows indicating locations of ORFs. Arrows have been colour coded, describing their predicted role (see key). The genome map was created with SnapGene 2.3.2.

A putative promoter with the consensus of AAAA(N3)TGTTGAC(N19)TATAAT was identified at 13 sites on the genome of CB7 (Supplementary information 1, Table S1). The sequence of this putative promoter resembles the consensus sequence of the sigma70 promoters of *Escherichia coli* (*E. coli*), possessing −10, −35 and the UP elements [26]. The locations of this promoter are heavily concentrated within a region containing a large number of ORFs encoding short hypothetical proteins (likely early gene region), with some located within a region possessing genes for a DNA helicase, DNA methylase and DNA polymerase (Figure 7). Based on the gene products downstream of these promoter sites, this would suggest that the promoter may play a role in early and possibly middle phase transcription. Moreover, analysis of genomes of *Cronobacter* phages CR3, CR8 and CR9 showed that this putative promoter is also present among their genomes, with 21, 21 and 13 sites being identified, respectively (Supplementary information 1, Tables S2–S4, and Figures S5–S7). Like CB7, the promoter is concentrated within a localized region containing a large number of ORFs encoding short hypothetical proteins (likely early gene region), as well as being located near a small number of ORFs with gene products involved in DNA replication and nucleotide metabolism (likely middle gene region). Identification of this promoter among these additional phages suggests that it may represent part of a conserved transcription strategy being employed among members of *Certrevirus*. Furthermore, 27 putative rho-independent terminators were also identified on the genome of CB7 (Supplementary information 1, Table S5).

### 2.5. DNA Replication, Methylation and Nucleotide Metabolism

Within the genome of CB7, ORFs for proteins associated with functions related to DNA replication were primarily found within gene region 2, while those for nucleotide metabolism were mostly found within gene region 4 (Figure 3). The phage was identified to have several genes encoding for proteins involved in DNA replication such as a DNA polymerase (CB7_49, 51, 53), DNA ligase (CB7_234), helicase (CB7_80) and a putative primase (CB7_73). There is also a homologue of recombination endonuclease VII of *Escherichia* phage T4 (CB7_214). In phage T4, this protein is involved in DNA mismatch repair and assists in DNA packaging by removing branched replicative DNA [27].

The phage genome also encodes proteins involved in nucleotide metabolism, including aerobic class I ribonucleotide reductase (RNR) subunits NrdA (CB7_195, 197) and NrdB (CB7_201, 203), whose role is the synthesis of deoxynucleoside triphosphates (dNTP) during aerobic conditions. In addition, the phage genome also encodes anaerobic class III ribonucleotide reductase subunits NrdG (CB7_187) and NrdD (CB7_188), which enable dNTP production under anaerobic conditions. These classes of RNRs have been identified among a number of phage genomes on GenBank [28]. Additionally, the RNR class I function of CB7 can potentially be supported by glutaredoxin (CB7_189) by allowing RNR reduction [29]. The phage is also capable of influencing the deoxythymidine monophosphate (dTMP) pool of the host by using deoxyuridine monophosphate(dUMP) with its thymidylate synthase (CB7_206) (IPR003669). It also has a ribose-phosphate pyrophosphokinase (CB7_242) (IPR005946) which forms the precursor phosphoribosyl pyrophosphate (PRPP), involved in the biosynthesis of purine and pyrimidine nucleotides. PRPP can also be used by CB7 with its nicotinamide phosphoribosyl transferase (CB7_244, 246) to produce nicotinamide mononucleotide, an intermediate in the production of coenzyme nicotinamide adenine dinucleotide (NAD).

The phage is also capable of influencing the tRNA pool of the host with a tRNA nucleotidyltransferase (CB7_179) (IPR012006), which synthesizes or repairs the 3’ terminal CCA sequence of tRNA molecules.

The identification of two kinds of DNA methylase enzymes on phage CB7 (CB7_63, 183) indicates that its genome is likely to contain methylated adenine and cytosine, given that it has a putative N-6 adenine-specific DNA methylase (DAM) (IPR002052) and DNA cytosine methylase (HHpred analysis: best hit; 5-cytosine DNA methyltransferase of *Entamoeba histolytica*, PDB accession no. 3QV2_A). Indeed, restriction digestion patterns of the genomic DNA of CB7 using ClaI does support that its DNA is likely to be DAM methylated (Supplementary information 1, Figure S8).

Comparison of CB7 to other members of the *Certrevirus* genus using ACT (TBLASTX) showed that the majority of the previously described proteins involved in DNA replication, methylation and nucleotide metabolism are shared by isolates across the genus (Supplementary information 1, Table S6). The exceptions to this are a homologue of the putative DNA cytosine methylase not shared with *Pectobacterium* phages ΦTE, DU_PP_I and DU_PP_IV. Another dissimilarity identified was that NrdD and NrdG of CB7 were only shared with ΦTE among the genus. These gene product variations suggest that there may be small differences between members of *Certrevirus* regarding DNA methylation and nucleotide metabolism, unless proteins for which a function could not be defined among these phages perform a similar role. Added comparison (using ACT with TBLASTX) between CB7 and type phages rV5 and PVP-SE1 revealed differences in gene product content. (Supplementary information 1, Table S7). Both type phages were found to share a homolog of the CB7 DNA ligase. Furthermore, phage rV5 was identified to not encode homologues of the CB7 tRNA nucleotidyl transferase and nicotinamide phosphoribosyl transferase.

### 2.6. Selfish Genetic Elements within the Genome of CB7

Homing endonucleases are mobile genetic elements consisting of genes that encode a protein with endonuclease activity that promotes the lateral transfer of their own encoding gene. These endonucleases recognize specific DNA sequences at which they initiate catalysis of DNA strand breakage, resulting in the insertion of a homing endonuclease encoding gene, due to DNA cellular mechanisms that rely on homologous recombination (the process being termed homing). Introns are segments of DNA that are removed from a mature mRNA post-transcription, while inteins are self-splicing protein elements that self-excise from a protein precursor with the concomitant post-translation ligation of C- and N-terminal segments called exteins. Homing endonucleases can be found associated with these elements or simply exist as free-standing genes [30]. A significant number of homing endonucleases have been characterized among phages with recognition sites that lie within genes related to DNA replication and metabolism [31], but they have also been identified to target genes related to virion structure [32,33].

On the genome of CB7, twenty one homing endonucleases of the HNH family (IPR003615, IPR029471) were identified (twenty HNHs confirmed with InterProScan, and one HNH identified with HHpred). Five of these HNH homing endonucleases were associated with an intron, with the remaining identified as free-standing genes (Table 3). ORFs for these homing endonucleases were found to cover approximately 7% of the genome of CB7.

A single intron with an HNH gene was found to be interrupting ORFs for the large terminase (CB7_1,3), ribonucleotide-diphosphate reductase (CB7_195, 197) and nicotinamide phosphoribosyl transferase (CB7_144, 246), with two introns with an HNH gene found to be interrupting ORFs of the DNA polymerase (CB7_49, 51, 53), with an additional HNH gene at the 5’ end of this gene product. These can be categorized as being group 1 introns as they possess homing endonucleases of the HNH family [30].

Reverse transcription PCR (RT-PCR) was performed to investigate the splicing of the previously described introns at the mRNA level. Total RNA was extracted from cells of *P. atrosepticum* strain DSM 30186 infected with phage CB7 at different time points (15 min, 30 min and 45 min). The resulting cDNA was then investigated for splicing using PCR with primers complementary to the 5’ and 3’ ends of the HNH endonuclease genes being examined and using CB7 genomic DNA as a control. The size of the resulting PCR product was then compared to that obtained from CB7 genomic DNA (Supplementary information 1, Figure S9). Splicing was shown to occur for introns containing an HNH for gene products of the large terminase (*CB7_3*), ribonucleotide reductase NrdB (*CB7_196*), nicotinamide phosphoribosyl transferase (*CB7_202*) and DNA polymerase (*CB7_50, 51*). This was supported by the appearance of PCR products derived from cDNA that were smaller than that those obtained from CB7 genomic DNA, indicating that splicing had occurred at the mRNA level causing the removal of HNH ORF sequences, thus resulting in smaller PCR products.

Under the RNA isolation conditions tested, no splicing occurred for DNA polymerase HNH *CB7_54*, situated at the 5’ end of ORF *CB7_53*. This is likely to be due to it being a free-standing HNH and not being part of an intron. However, HNH *CB7_245* is associated with an intron that interrupts the ORFs of nicotinamide phosphoribosyl transferase and was not found to be removed by splicing at the mRNA level. The existence of introns with an HNH that do not splice post-transcription (like that of the intron with HNH *CB7_245*) has been previously described. *Aeromonas* phage Aeh1 anaerobic ribonucleotide reductase subunit NrdA is split by an intron that contains an HNH endonuclease (*mobE*) and does not splice post-transcription. This intron causes NrdA to be translated as two separate peptides that associate with each other post-translation, along with the ribonucleotide reductase NrdB subunit while still retaining activity [33]. This may also be the case for the nicotinamide phosphoribosyl transferase of CB7. The above HNH splicing results of CB7 were further confirmed by preforming Sanger sequencing on the PCR products derived from cDNA.

Three inteins were identified among the ORFs of CB7, namely the putative helicase (CB7_80), the ribonucleotide reductase NrdB (CB_197) and NrdA (CB7_201) (Table 3). Furthermore, the inteins of ORFs CB7_80 and CB7_201 were found to contain the homing endonuclease of the LAGLIDADG family (IPR004860). Experiments were not conducted to determine the splicing nature of these elements within the timeframe of this study.

In total, twenty-three homing endonucleases were identified to be present on the genome of CB7. This is one of the largest quantities of homing endonucleases identified on a phage genome described in the literature to date, greater than that described for *Escherichia* phage T4 containing fifteen [34]. Most of the homing endonucleases of CB7 are shared with *Pectobacterium* phage ΦTE, and less so with the *Cronobacter* phages of the *Certrevirus* genus (Table 3). *Cronobacter* phage CR3 is only predicted to possess two HNH homing endonucleases. The presence of a high number of homing endonucleases appears to be a common trend among many phage types infecting bacteria of the SRE group [8,11,13].

### 2.7. Structural Proteome Analysis of Phage CB7 Particles

The majority of ORFs identified to encode proteins involved in the morphogenesis of the virion of CB7, including the large terminase (CB7_1, 3) which plays a role in the capsid packaging of genomic DNA, are located within gene region 1 of the CB7 genome. One exception was the ORF of structural protein CB7_55, which is situated in gene region 2 (Figure 3). ESI-MS/MS analysis was conducted on purified virions of CB7, and this is the first phage of the *Certrevirus* genus to have this analysis performed. A total of twenty-six proteins were identified to form its virion (Table 4). Those for which a function could be inferred were putative tail proteins (CB7_17, 18, 23, 26, 31), tail fibre proteins (CB7_10, 36), tail baseplate proteins (CB7_27, 28, 29, 30) and capsid proteins (CB7_4, 5, 7, 8). In silico analysis of the other eleven structural proteins identified failed to find a putative role.

Putative structural protein CB7_32 was identified to encode a tail assembly protein due to it possessing a phage T4 gp38 tail assembly domain (IPR003458). This was not detected by mass spectrometry (MS) analysis. Protein gp38 of phage T4 acts as a chaperone protein in the assembly of the long tail fibre and is not present in the mature phage particle. This may also be the case for the gene product of CB7_32.

The proteins that may be involved in host recognition are CB7_10 and CB7_36, as they may play a role in the structure of phage tail fibres. Gene product CB7_10 possesses a collagen domain (IPR008160), and such domains are commonly associated with phage tail fibre proteins [35]. Additionally, the putative tail collar protein CB7_31 may also play a role in host cell attachment, given that it possesses an Ig domain (IPR003343). Such domains commonly occur on several phage virion proteins such as the tail fibre, the baseplate wedge initiator, the major tail and major capsid proteins. It is believed that this domain may interact weakly with carbohydrates present on the host cell surface [36].

Comparison between phages of the *Certrevirus* genus shows that the majority of the identified structural proteins of phage CB7 are shared among members. Exceptions to this are CB7_55, with homologues of this protein only shared with phages outside the *Certrevirus* genus, such as *Erwinia* phages PhiEaH1 (accession no. YP_009010139) and vB_Eam_Stratton (accession no. ANZ50590). Also, the structural protein CB7_251 only has a homologue with phage ΦTE (Supplementary information 1, Table S8).

When type phages rV5 and PVP-SE1 were first described, the number of proteins that were identified to form the virion of these phages were sixteen and thirty-six proteins, respectively. MS analysis of both of these virions allowed the confirmation of six (for rV5) and twenty-five (for PVP-SE1) of these proteins [37,38]. Comparisons of these two phages with CB7 show that they share twenty structural proteins, with minimal similarity occurring between several proteins predicted to play roles in tail fibre structure (Supplementary information 1, Table S9). It has been described that host cell binding receptors of *Cretrevirus* phages ΦTE and CR3 are the flagella of the host cell, while lipopolysaccharide (LPS) is believed to be the host cell binding receptor of other phages belonging to the subfamily *Vequintavirinae* [14,16,39] The lack of homology among these tail fibre proteins likely reflects differences among the host cell receptors recognized by phages belonging to different genera of *Vequintavirinae.*

### 2.8. Cell Wall-Degrading Enzymes and Cell Lysis Proteins

Peptidoglycan-degrading enzymes are used during the initial stages of phage infection to penetrate the host cell wall during injection of phage DNA (virion-associated lysins). They are also employed during host cell lysis at the end of phage lytic cycle (endolysins) [40]. Three potential peptidoglycan-degrading enzymes were identified in the genome of CB7 (Supplementary information 1, Table S10). CB7_28 is a putative virion-associated lysin and HHpred analysis of this protein and its homologues among *Certrevirus* and phages rV5 and PVP-SE1 showed that they are homologous of gp25 of *Escherichia* phage T4 (using CB7_28; best hit gp25-like lysozyme of *Geobacter sulfurreducens,* PDB accession no. 2IA7_A). This protein of phage T4 forms part of the phage’s baseplate and possesses acidic lysozyme activity [41]. CB7_83 is predicted to be a putative cell wall hydrolase (N-acetylmuramyl-L-alanine amidase) resembling SleB, a protein in *Bacillus subtilis* responsible for the hydrolysis of the spore cortex during germination (IPR011105). Homologues of CB7_83 were identified among members of all genera within the *Vequintavirinae* subfamily. A homologue of this protein in *Cronobacter* phage CR3 (namely, CR3_087) has been implicated in host lysis [16]. Furthermore, the protein was identified to possess a signal peptide sequence. Attempts to express this protein recombinantly in *E. coli* show the protein to be toxic on expression (data not shown). CB7_190 is a potential peptidase (IPR009045), with HHpred analysis showing homology to the endolysin of *Escherichia* phage T5 (L-alanyl-D-glutamate peptidase, PDB accession no. 2MXZ). This putative peptidoglycan-degrading enzyme has only been identified in *Pectobacterium* phages CB7 and ΦTE (phiTE_147) within the *Certrevirus* genus, whereas the other genus members encode a putative peptidoglycan-degrading enzyme of different origin and possibly enzymatic activity (IPR023346).

A putative Rz/Rz1 spanin pair (CB7_252/253) was also identified: these proteins are conserved among members of the *Certrevirus* genus as well as phages rV5 and PVP-SE1 (Supplementary information 1, Table S11). They play a role in the destruction of the outer membrane of Gram negative cells during host cell lysis allowing progeny phage release at the end of the infection. Like other spanin proteins, the CB7_252/253 are typical examples, one possessing an N-terminal transmembrane domain and the other possessing a lipoprotein signal sequence [42] The ORFs of these consist of separate coding sequences where the stop codon of the Rz gene overlaps with the start codon of the Rz1 gene. This gene arrangement is in common with *Escherichia* phage T4 [42]. For CB7, rV5 and PVP-SE1, this spanin protein pair is not associated with a classic lysis cassette as their genes are located next to those of the large terminase. A similar gene arrangement has also been seen in a number of Podoviruses such as *Vibrio* phage VP4 and Enterobacteria phage SP6 [42].

Another class of proteins associated with host lysis are holins. These create openings in the host cytoplasmic membrane, allowing the phage endolysin to access cell wall peptidoglycan [43]. CB7 (including other members of *Certrevirus*) does not appear to have a classic lysis cassette, in which all the lysis genes are in proximity to each other, and this makes the identification of candidate holins difficult. However, in the case of CB7, a hypothetical membrane protein (65 amino acid residues in length) was observed, whose ORF overlaps that of the putative SleB-like protein CB7_83. This protein is predicted to possess two transmembrane domains where its N- and C-termini are situated in the host cell cytoplasm, making it a strong candidate for a class II holin (CB7_82) [43]. A similar protein is found among a number of other phages of the *Certrevirus* genus (phage CR9 being an exception) downstream of their SleB-like protein (Supplementary information 1, Table S11).

Phage CB7 does not appear to have homologues of rIIA and rIIB, which are present in rV5 and PVP-SE1. The function of these genes is not well understood, but they are thought to have an influence on the regulation on host lysis relating to membrane integrity and energetics [44]

## 3. Discussion

The *Certrevirus* ICTV-defined genus was the first established genera of phages described to infect *P. atrosepticum*, with *Munavirus* being the second [13]. However, the coming years should see the establishment of additional genera, with at least one other phage type known to infect this SRE species [11]. The categorization of phage within defined genera should assist in the understanding of their shared biology and indeed in their selection as biocontrol agents.

Phylogenetic analysis in this study allowed the positioning of *Pectobacterium* phage CB7 within *Certrevirus* (Table 2, Figure 4, Figure 5, Figure 6 and Supplementary information 1; Figure S4). Examination of this phage and comparative analysis with other members within the genus show they possess a conserved transcriptional genome organization, involving the use of a promoter with elements resembling that of the *E. coli* sigma70 promoter (Figure 7, Supplementary information 1, Tables S1–S4). Moreover, these phages appear to possess conserved strategies of DNA replication, DNA metabolism, host lysis and virion structure as indicated by their shared protein content associated with these processes. However, there also appears to be subtle diversity within the genus, considering the absence of protein homologs involved in DNA methylation, nucleotide metabolism, virion structure and those involved in peptidoglycan degradation among some members (Supplementary information 1, Tables S6, S8, S11).

Of the twenty-six structural proteins identified to form the virion of phage CB7 (Table 4), twenty were found to be shared with the type phages rV5 and PVP-SE1 of *Vequintavirus* and *Seunavirus*, respectively (Supplementary information 1, Table S9). The differences within protein content among these phages are concentrated within those proteins predicted to form the tail fibre. Such differences likely reflect the adaption of the tail fibre of these phages to allow the recognition of their respective host cell receptors. Both *Pectobacterium* phage ΦTE and *Cronobacter* phage CR3 share a flagellum-dependent recognition of their respective host bacterium, suggesting this mechanism is likely shared among *Certrevirus* members. *Pectobacterium* phage ΦATI described by Evans et al. [45] employs flagellum receptor specificity and is quite likely a *Certrevirus,* as currently available partial genome sequences (accession nos. FN396585, FN396583 and FN396595) possess significant homology to that of *Certrevirus* phages. It was reported that mutants of *P. atrosepticum* resistant to phage ΦATI infection due to defective flagella had reduced virulence in potato tuber rot assays [46]. Such reduced virulence resulting from phage application would be a desirable outcome were host resistance to develop. However, a member of this genus *Pectobacterium* phage ΦTE has been shown to be capable of causing generalized transduction [14]. Both phages CB7 (Supplementary information 1, Figure S3) and ΦTE have been identified to possess circularly permuted genomes, and the feature is likely shared with other members of *Certrevirus* [14]. Due to the physical nature of these phage genomes, it is likely that they employ a headful packaging strategy. Such strategies are known to have a high occurrence of generalized transduction [20,47]. Such a genomic feature could hypothetically contribute to the spread of virulence factors among different strains of the bacterial host, were transduction to occur after application of the phages for biocontrol strategies [48]. On the other hand, T4-like phages with a similar packaging strategy have been deemed suitable for biocontrol applications suggesting that it is not a major consideration [49,50].

For the application of phages for biocontrol purposes, the possession of a wide host range against a pathogen of interest is deemed a desirable feature [48]. In this regard, the selection of phage CB7, and possibly other *Certrevirus* phages for the biocontrol of *P. atrosecpetium* is likely restricted. As the phage was found to only form plaques on only 26% (5/19) of tested strains (Table 1), greater host range was previously reported among N4-like phages that infect this bacterium, which were found to plaque on up to 63% (12/19) of examined strains [11]. However, it was not as limited as *Phimunavirus,* with a phage of this genus only forming plaques on 16% (3/19) of tested strains [13]. Nevertheless, the utility of phages of *Certrevirus* infecting this phytopathogen cannot be completely ruled out. As when designing a phage cocktail for biocontrol, it should be composed of differing phages that recognize varying host cell receptors to limit the emergence of phage resistance [48]. Currently, data on the host cell receptors recognized by the different phage of *P. atrosepticum* is highly limited; greater effort is now needed to determine these elements so as to enable the optimal design of phage cocktails against this bacterium.

As already stated, 21 of the ORFs of phage CB7 encode HNHs, with these covering 7% of its total genome sequence. With six of these forming introns among the large terminase, DNA polymerase, ribonucleotide reductase NrdA and NrdB and nicotinamide phosphoribosyl transferase genes of the phage (Table 3). The type of genes targeted by these HNH-associated introns are quite typical, having been seen among other phage types infecting other hosts, such as those of *Kayvirus* infecting *Staphylococcus* [51]. However, one must ask, why would a phage like CB7 possess so many of these elements? Surely, their presence in this quantity could only have a negative impact, unless they play some sort of regulatory mechanism or somehow enable infection. Indeed, it has been shown that HNH-associated introns accompanying ORFs of the large terminase can play a role with the stimulation of protein function with the packaging of genomic DNA into phage capsids. [33] However, even if this is the case with all HNH-associated introns, playing a role with the stimulation of function of the proteins of whose genes they are embedded, what about the free-standing HNHs? What pressure could cause their accumulation in a genome like that of phage CB7? This is a question that merits further attention.

## 4. Materials and Methods 

### 4.1. Phage Isolation

CB7 was isolated using an enrichment method. Five grams of soil was weighed and placed into 30 mL of LB broth, together with 300 µL of a mixed overnight culture of *P. atrosepticum* strains (DSM 18077, DSM 30184, DSM 30185, and DSM 30186) and then incubated for 18 h at 25 °C. This was centrifuged to pellet soil matter with the supernatant then being filtered (0.45 µm pore-size filter, Sarstedt, Nümbrecht, Germany). The supernatant was spotted (10 µL) onto LB overlays that were seeded with different strains of *Pectobacterium*. CB7 was isolated by picking individual plaques and then re-plated and re-isolated to ensure purity, using *P. atrosepticum* DSM 30186 [52].

### 4.2. Host Range and General Characterization

The host range of the phage was tested by spotting serial dilutions (neat to dilution 10^−9^) of a phage suspension onto LB overlays seeded with the appropriate bacterial host, as described previously [53] with two biological repeats being preformed. Bacteria strains used in host study are listed in Supplementary information 1, Table S12.

A similar approach to the single-step growth curve assay described previously was used [54,55]. The host bacteria (strain DSM 30186) were grown to an OD_600_ of 0.20–0.23 (approximately 1 × 10^8^ colony forming units (CFU)/mL), followed by centrifugation of 2 mL in a microfuge to pellet bacteria. The pellet was resuspended in 1 mL of phage suspension to yield an approximate multiplicity of infection (MOI) of 5 × 10^−4^ following incubation at 25 °C for one minute. This was then centrifuged to pellet bacteria, and the supernatant was removed, thus separating bound phages from unbound phages. The bacterial pellet with bound phage was then resuspended in 10 mL of LB and incubated aerobically in a water bath at 25 °C with agitation at 60 rpm. At 5 min intervals, aliquots were removed to measure phage titre by the overlay method. Based on the number of plaque-forming units (PFU)/mL, the latent period and burst size were determined. The burst size was calculated by dividing the average PFU/mL during the latent period by the average PFU/mL value after phage titre had plateaued after the initial increase. Three biological repeats were conducted with the mean result used for calculations.

Phage stability was tested by incubating phage suspension of 10^6^ PFU/mL in SM buffer (50 mM Tris-HCl pH 7.5, 100 mM NaCl, 8 mM MgSO_4_) at different temperatures for one hour and phage suspension in pH buffer ranging from 2 to 12 (10 mM trisodium citrate, 10 mM boric acid, and 150 mM KCl, adjusted with NaOH or HCl) for 24 h [8]. Stability in chloroform was tested by shaking in 17% [v/v] chloroform for one hour. These assays were performed with two biological repeats with three technical repeats; the results of a single biological replicate are reported.

### 4.3. Cesium Chloride Gradient Purification

Isopycnic centrifugation through caesium chloride (CsCl) gradients was performed, as previously described [56], with a number of modifications. A high-titer phage lysate (>1 × 10^9^ PFU/mL) was precipitated using polyethylene glycol (15% w/v PEG8000, 1 M NaCl) at 4 °C overnight and centrifuged, after which the pellet was resuspended in TMN buffer (10 mM Tris-HCl pH 7.4, 10 mM MgSO_4_·7H_2_O, 0.5M NaCl) and, where necessary, a chloroform phase separation step (1:1) was conducted to remove debris. The resulting phage preparation was placed onto a CsCl step gradient composed of 1.3, 1.5, and 1.7 g/mL layers and spun in a 100 Ti rotor (Beckman Coulter, Brea, CA, USA) at 200,480× *g* for 3 h at 4 °C. Resulting phage bands were collected and subjected to dialysis with two changes of Tris-HCl buffer (10 mM, pH 7.5) at 4 °C.

### 4.4. Transmission Electron Microscopy

Phages adsorbed to freshly prepared ultra-thin carbon film were i) fixed with 1% (v/v) EM-grade glutaraldehyde (20 min), ii) negatively stained with 1% (w/v) uranyl acetate, and iii) subsequently analyzed using a Tecnai 10 transmission electron microscope (FEI Thermo Fisher, Eindhoven, the Netherlands) at an acceleration voltage of 80 kV. Digital micrographs were acquired with a MegaView G2 CCD-camera (EMSIS, Muenster, Germany).

### 4.5. DNA Isolation, Restriction and Sequencing

DNA extraction was performed as previously described [57]. CsCl-purified phage particles were treated with DNase and RNase, followed by treatment with 10% sodium dodecyl sulfate (SDS) and proteinase K followed by DNA extraction with phenol: chloroform: isoamyl alcohol (25:24:1 v/v) and chloroform: isoamyl alcohol (24:1 v/v). DNA samples were digested with BamHI and SspI, according to manufacturer’s protocols (New England BioLabs, Ipswich, MA, USA). The digested DNA was analyzed by agarose gel electrophoresis.

Prior to sequencing, DNA quality and quantity were estimated using both a Nanodrop (ND-1000, Thermo Fisher, Waltham, MA, USA) and by visualization after agarose gel electrophoresis. Genomic sequencing was outsourced to the Centre for Genomic Research at the University of Liverpool, Liverpool, UK. Illumina MiSeq system (Illumina, San Diego, CA, USA), with a TruSeq DNA Nano LT library sample preparation kit for library preparation. Library quality was assessed using the Agilent Bioanalyzer (Agilent Technologies, Santa Clara, CA, USA) and Qubit measurements prior to being sequenced with paired-end reads of 2 × 250 bp. Reads were assembled using Spades genome assembler v.3.10 [58].

### 4.6. BAL-31 Nuclease Treatment of Genomic DNA

A total of 40 μg of CB7 DNA was digested with BAL-31 (0.5 units per μg) (New England Biolabs) at 30 °C. Over a series of time intervals, aliquots were taken, treated with ethylene glycol-bis(β-aminoethyl ether) (EGTA) for BAL-31 deactivation and then subjected to digestion with restriction enzyme BglII CB7 at 37 °C (New England Biolabs) [59]. Resulting genomic DNA was visualized by agarose gel electrophoresis.

### 4.7. Bioinformatic Analysis

Open reading frames (ORFs) of CB7 were predicted with GLIMMER [60] and GenemarkS [61]. Functional inferences for predicted ORF gene products were obtained by searches conducted using BLASTp (http://blast.ncbi.nlm.nih.gov/Blast.cgi?PAGE=Proteins; [62]), Pfam (http://pfam.xfam.org/search#tabview=tab1; [63]), InterProScan (https://www.ncbi.nlm.nih.gov/pmc/articles/PMC3998142/; [64]) and HHpred (https://toolkit.tuebingen.mpg.de/#/tools/hhpred; [65]). Transmembrane domains and lipoprotein cleavage signal were identified using TMHMM v.2 (http://www.cbs.dtu.dk/services/TMHMM/; [27]) and LipoP v.1 (http://www.cbs.dtu.dk/services/LipoP/; [66]), respectively. Translated ORFs from phage φM1 were searched against hidden Markov model profiles downloaded from the pVOGs database (ref) using hmmscan (version), with an E-value cut off of 10^−5^. Screening for antibiotic resistant genes was achieved with ABRicate (available at https://usegalaxy.eu/). The molecular weight of the predicted ORFs was estimated using the batch protein molecular weight determination of the sequence manipulation suite (http://www.bioinformatics.org/sms2/protein_mw.html). The presence of transfer RNA (tRNA) genes was investigated with the use of tRNAscan-SE (http://lowelab.ucsc.edu/tRNAscan-SE/; [67] and ARAGORN (http://130.235.46.10/ARAGORN/; [30]). The circular genome map of CB7 was drawn using GCview (http://stothard.afns.ualberta.ca/cgview_server/; [25]). Potential Rho-independent terminators were identified using ARNold (http://rna.igmors.u-psud.fr/toolbox/arnold; [68]) with Mfold QuikFold (http://unafold.rna.albany.edu/?q=DINAMelt/Quickfold; [69]) using RNA energy rules 3.0 to verify predictions. Potential promoters were identified by submitting up stream sequences (100 bp) of predicted genes to Multiple Em for Motif Elicitation (MEME) (http://meme-suite.org/tools/meme; [70]).

### 4.8. Comparative Genomics

To determine shared proteins among phage proteomes, CoreGenes 3.5 was used (http://gateway.binf.gmu.edu:8080/CoreGenes3.5/custdata.html; [71]). The linear genomic comparison maps were created with the use of either BLASTN or TBLASTX to determine homology, and then visualized with EasyFig [72]. Artemis Comparison Tool (ACT) was used to identify feature variations between phage genomes of *Vequintavirinae*, with homology assessed by TBLASTX [73]. Phylograms were generated based on the amino acid sequence of the major capsid protein, larger terminase and DNA polymerase of phage CB7 and 17 members of *Vequintavirinae*. Trees were created using MEGA7 [74], applying MUSCLE for sequence alignment [75] with the construction of phylograms using the maximum likelihood (ML) method based on the Whelan and Goldman substitution model [76], with the robustness of the trees assessed by bootstrapping (1000). VICTOR was employed using all pairwise comparisons of the amino acid sequences (same phages as described previously), applying the Genome-BLAST Distance Phylogeny (GBDP) method [77], under settings recommended for prokaryotic viruses [78]. The resulting intergenomic distances (including 100 replicates each) were used to infer a balanced minimum evolution tree with branch support via FASTME including SPR post-processing [79] for each of the formulas D0, D4 and D6, respectively. The trees were rooted at the midpoint [80] and visualized with FigTree (http://tree.bio.ed.ac.uk/software/figtree/). Taxon boundaries at the species, genus, subfamily and family level were estimated with the OPTSIL program [81], the recommended clustering thresholds [78] and an F value (fraction of links required for cluster fusion) of 0.5 [82]. The resulting phylogram was manipulated with iTOL [83]. The heat map comparing the genomes of phage CB7 and 17 members of *Vequintavirinae* was generated using Gegenees utilizing TBLASTX, with accurate parameters (fragment length: 200 bp; step size: 100 bp, threshold set to 0%) [84].

### 4.9. Comparative Genomics

A similar approach was taken as previously described [85,86]. Freshly grown host bacteria (strain DSM 30186) at an OD_600_ of 0.20–0.23 was infected with phage CB7 at an MOI of 5 × 10^−4^, in the same manner as described for the single-step growth curve assay above. At 15, 30, and 45 min intervals, 1 mL of infected cells was pelleted and resuspended in phosphate buffer saline (PBS). Next, total RNA was extracted using the High Pure RNA Roche extraction kit (Roche, Basel, Switzerland) and subsequently, RT-PCR was performed using the cDNA synthesis kit (Bioline, London, UK) according to the manufacturer protocol. PCRs were performed using RedTaq ReadyMix (Sigma-Aldrich, St. Louis, MO, USA) to amplify products using primers specific to regions situated at the 5′ and 3′ ends of HNH endonuclease(s) being examined, with cDNA and CB7 genomic DNA as controls. These products were then inspected by agarose gel electrophoresis and Sanger sequencing for the presence of splicing. Primers used in PCRs are detailed in Supplementary information 1, Table S13.

### 4.10. Mass Spectrometric Analysis of the Phage CB7 Virion Proteome

Phage capsid proteins were extracted from high-titer CsCl-purified phage (5 mL, titre of 1 × 10^10^ PFU/mL) using chloroform:methanol extraction (1:1:0.75, v/v/v). The resulting protein pellet was resuspended in loading buffer (1% SDS, 6% sucrose, 100 mM dithiothreitol, 10 mM Tris pH 6.8, 0.0625% w/v bromophenol blue) and heated to 95 °C for 5 min to resuspend the pellet. This was subsequently loaded onto a 12% SDS-PAGE gel, after which gel electrophoresis was conducted. The resulting gel was then stained using Gelcode™ Blue Safe Protein Stain (Thermo Fisher) to visualize virion proteins. Gel fragments were trypsinized and peptide extracts analyzed using ESI-MS/MS, as described previously [87].

### 4.11. Accession Number

The genome of *Pectobacterium* phage CB7 was deposited on GenBank under accession number KY514263.

## Figures and Tables

**Table 1:** Host range of *Pectobacterium* phage CB7 on 31 strains of soft rot *Enterobacteriaceae* and others as determined by spot testing with a serial dilution of phage.

Species	Strain	Sensitivity
*Pectobacterium atrosepticum*	DSMZ 18077 (type strain)	-
	DSMZ 30184	-
	DSMZ 30185	+
	DSMZ 30186	+ *
	CB BL1-1	-
	CB BL2-1	+
	CB BL3-1	+
	CB BL4-1	+
	CB BL5-1	-
	CB BL7-1	-
	CB BL9-1	-
	CB BL11-1	-
	CB BL12-2	-
	CB BL13-1	-
	CB BL14-1	-
	CB BL15-1	-
	CB BL16-1	-
	CB BL18-1	-
	CB BL19-1	-
*Pectobacterium carotovorum* subsp. *carotovorum*	DSMZ 30168 (type strain)	-
	DSMZ 30169	-
	DSMZ 30170	-
	CB BL19-1-37	-
*Dickeya chrysanthemi* bv. *chrysanthemi*	LMG 2804	-
*Dickeya dianthicola*	PD 482	-
	PD 2174	-
	GBBC 1538	-
*Dickeya solani*	sp. PRI 2222 (D36)	-
	LMG 25865 (D10)	-
	GBBC 1502	-
	GBBC 1586	-
*Erwinia amylovora*	LMG 2024	-
	GBBC 403	-
*Erwinia mallotivora*	LMG 1271	-

Results recorded as +, sensitive; −, no infection; * host strain of phage.

**Table 2:** Properties of the five phages currently forming *Certrevirus*, and *Pectobacterium* phages DU_PP_I and DU_PP_V and CB7. Each of the seven phages was compared to the type phage CR3.

Phage	Accession No.	Genome Size (bp)	G+C Content (%)	ORFs	tRNA	Identity (%) *	Shared Proteins (%) **
*Cronobacter* phage CR3	JQ691612	149,273	50.9	265	18	100	100
*Cronobacter* phage CR8	KC954774	149,162	50.8	269	17	94	80
*Cronobacter* phage CR9	JQ691611	151,924	50.6	281	17	70	89
*Cronobacter* phage PBES 02	KT353109	149,732	50.7	270	14	64	90
*Pectobacterium* phage DU_PP_I	MF979560	144,959	50.1	267	8	73	80
*Pectobacterium* phage DU_PP_IV	MF979563	145,233	50.3	268	8	73	80
*Pectobacterium* phage vB_PatM_CB7	KY514263	142,778	50.1	253	1	64	70
*Pectobacterium* phage ΦTE	JQ015307	142,349	50.1	242	2	62	63

* DNA identity in comparison to CR3 using BLASTN. ** number of homologous proteins in comparison to CR3 using CoreGenes.

**Table 3:** Inteins and homing endonucleases (free standing or intron associated) identified in the genome of *Pectobacterium* phage CB7, using ACT (TBLASTX) for comparison with *Pectobacterium* phage ΦTE and *Cronobacter* phage Cr3.

ORF/Associated ORF	Selfish Genetic Element	Homing Endonuclease Family	Gene Product Function of Targeted Gene	Does Splicing Occur (mRNA Level)?	Shared with ΦTE	Shared with Cr3
CB7_2	intron associated with homing endonuclease	HNH	Large terminase (CB7_1,3)	Yes	Shared	No
CB7_38	free-standing homing endonuclease	HNH	_	_	Shared	No
CB7_48	free-standing homing endonuclease	HNH	_	_	No	No
CB7_50	intron associated with homing endonuclease	HNH	DNA polymerase (CB7_49, 51)	Yes	Shared	No
CB7_52	intron associated with homing endonuclease	HNH	DNA polymerase (CB7_51, 53)	Yes	Shared	No
CB7_54	free-standing homing endonuclease	HNH	_	No	shared	No
CB7_62	free-standing homing endonuclease	HNH	_	_	No	No
CB7_80	intein associated with homing endonuclease	LAGLIDADG	Putative helicase (CB7_80)	_	Shared	_
CB7_109	free-standing homing endonuclease	HNH	_	_	Shared	No
CB7_143	free-standing homing endonuclease	HNH	_	_	Shared	No
CB7_180	free-standing homing endonuclease	HNH	_	_	Shared	No
CB7_182	free-standing homing endonuclease	HNH	_	_	Shared	No
CB7_196	intron associated with mobile genetic element	HNH	Ribonucleotide reductase NrdB, part 1 and 2 (CB7_195, 197)	Yes	No	No
CB7_197	intein with no homing endonuclease	_	Ribonucleotide reductase NrdB, part 2 (CB_197)	_	Shared	_
CB7_201	intein associated with homing endonuclease	LAGLIDADG	Ribonucleotide reductase NrdA, part 1 (CB7_201)	_	Shared	_
CB7_202	intron associated with homing endonuclease	HNH	Ribonucleotide reductase NrdA (CB7_201, 203)	Yes	Shared	No
CB7_212	free-standing homing endonuclease	HNH	_	_	Shared	No
CB7_219	free-standing homing endonuclease	HNH	_	_	Shared	No
CB7_220	free-standing homing endonuclease	HNH	_	_	Shared	Shared
CB7_221	free-standing homing endonuclease	HNH	_	_	Shared	No
CB7_230	free-standing homing endonuclease	HNH	_	_	Shared	No
CB7_243	free-standing homing endonuclease	HNH	_	_	Shared	No
CB7_245	intron associated with homing endonuclease	HNH	Nicotinamide phosphoribosyl Transferase (CB7_245)	No	Shared	No
CB7_249	free-standing homing endonuclease	HNH	_	_	No	No

**Table 4:** Results of tandem mass spectrometry of proteins of the *Pectobacterium* phage CB7 virion.

ORF	Predicted Function	pVOG	Protein Molecular Weight (kDa)	No. of Unique Peptides	Sequence Coverage %
CB7_4	putative portal protein	VOG1356	55.37	35	75
CB7_5	putative prohead core protein protease	VOG4722	21.35	2	16
CB7_6	unknown structural protein	VOG1355	40.57	3	14
CB7_7	putative head stabilization/decoration protein	VOG5255	15.6	16	96
CB7_8	putative major head protein	VOG0976	37.6	31	93
CB7_10	putative tail fibre protein	VOG9955	111.95	39	46
CB7_11	unknown structural protein	VOG5488	24.54	7	30
CB7_12	unknown structural protein	_	17.19	8	47
CB7_13	unknown structural protein	VOG5152	20.14	4	31
CB7_15	unknown structural protein	VOG1428	16.58	5	44
CB7_16	unknown structural protein	VOG5266	26.47	3	16
CB7_17	putative tail sheath protein	VOG1352	50.69	27	90
CB7_18	putative tail tube protein	VOG4699	17.2	6	45
CB7_23	putative tape measure protein	VOG10480	87.72	30	41
CB7_24	unknown structural protein	VOG1956	31.67	5	24
CB7_25	unknown structural protein	VOG1433	14.38	1	15
CB7_26	putative tail protein	VOG1348	36.5	1	5
CB7_27	putative baseplate protein	VOG0573	26.42	9	63
CB7_28	putative tail lysozyme	VOG4550	20.45	1	8
CB7_29	putative baseplate wedge protein	VOG4691	54.37	10	22
CB7_30	putative baseplate protein	VOG4620	24.2	10	56
CB7_31	putative tail collar protein	VOG8134	59.21	19	46
CB7_36	putative tail fibre protein	VOG4546	81.89	19	34
CB7_37	unknown structural protein	_	43.48	4	19
CB7_55	unknown structural protein	VOG7652	26.53	4	24
CB7_251	unknown structural protein	_	19.68	1	10

## Supplementary Materials

The following are available online at https://www.mdpi.com/2079-6382/9/6/352/s1; Figure S1: Genomic DNA of *Pectobacterium* phage CB7 digested with restriction enzyme BglII and SspI; Figure S2: *Pectobacterium* phage CB7 plaque morphology on 0.4% w/v LB overlay using host strain *P. atrosepticum* DSM 30186 after a 12 h incubation; Figure S3: Treatment of *Pectobacterium* phage CB7 genomic DNA with BAL-31, Figure S4: Phylograms constructed using the large terminase, major head and DNA polymerase of *Pectobacterium* phage CB7 and homologs of 17 phages of the *Vequintavirinae* subfamily; Figure S5. Genome map of *Cronobacter* phage CR3 (accession no. JQ691612), showing locations of a putative sigma70-like promoter; Figure S6. Genome map of *Cronobacter* phage CR8 (accession no. KC954774) showing locations of a putative sigma70-like promoter; Figure S7. Genome map of *Cronobacter* phage CR9 (accession no. JQ691611) showing locations of a putative sigma70-like promoter; Figure S8. Genomic DNA of *Pectobacterium* phage vB_PatM_CB7, digested with restriction enzyme ClaI; Figure S9. Gel images showing PCR products demonstrating splicing of intron-associated homing endonucleases associated with *Pectobacterium* phage CB7 using primers complementary to the 5’ and 3’ ends of the ORFs; Table S1: Annotation of the genome of *Pectobacterium* phage CB7; Table S2: Putative sigma70–like promoter sequences detected in the genome of *Pectobacterium* phage CB7; Table S3: Putative sigma70-like promoters detected in the genome of *Cronobacter* phage CR3; Table S4: Putative sigma70-like promoters detected in the genome of *Cronobacter* phage CR8; Table S5: Putative sigma70-like promoters detected in the genome of *Cronobacter* phage CR9; Table S6: High ΔG rho-independent terminators predicted in the genome *Pectobacterium* phage CB7, identified using ARNold and QuikFold; Table S7: Comparison of potential gene products of CB7 identified to be involved in DNA replication, methylation and nucleotide metabolism members of *Certrevirus* members, generated using ACT (TBLASTX); Table S8: Comparison of potential gene products of CB7 identified in DNA replication, methylation and nucleotide metabolism with that of *Escherichia* phage rv5 and Salmonella phage PVP-SE1, using ACT; Table S9: Shared structural proteins of phages of *Certrevirus* determined using ACT (TBLASTX); Table S10: List of primers used for the creation of PCR products from cDNA and genomic DNA of Pectobacterium phage CB7, used for the investigation of HNH endonuclease splicing. Table S11: Shared structural proteins (using currently available annotation) of phages of *Vequintavirinae*; Table S12: Putative cell wall-degrading and cell lysis proteins of phages in the subfamily *Vequintavirinae*; Table S13: Bacteria strains used in the isolation and the host range testing of *Pectobacterium* phage CB7.
